# SOCIAL INTEGRATION, COGNITION, AND FUNCTIONAL OUTCOME IN OLDER ADULTS FOLLOWING TRAUMATIC BRAIN INJURY

**DOI:** 10.1093/geroni/igad104.2383

**Published:** 2023-12-21

**Authors:** Wonkyung Jung, Jeanne Hoffman, Eeeseung Byun, Hilaire Thompson

**Affiliations:** Johns Hopkins University, Baltimore, Maryland, United States; University of Washington, Seattle, Washington, United States; University of Washington, Seattle, Washington, United States; University of Washington, Seattle, Washington, United States

## Abstract

Persons aged 65 and older have the highest rates of traumatic brain injury (TBI), and this injury is known to be related to significant, persistent functional changes. What is not known is what factors may mediate these changes. The current study aimed 1) to examine interrelationships among social integration, functional outcome, and cognition in older adults at years 1, 2, and 5 post-traumatic brain injury (TBI) and 2) to examine if functional outcome mediates the concurrent and longitudinal relationships between social integration and cognitive function post-TBI in older adults. This is a secondary analysis of data obtained from Traumatic Brain Injury Model System National Database (TBIMS-NDB). Included subjects from the database were persons aged 65 years and older at the time of injury (N=1469). We used longitudinal mediation analysis to address study aims. We found that social integration had a direct effect on functional outcome and cognition at 1, 2, and 5-years post-TBI. Functional outcome had a positive direct effect on concurrent cognitive function. Regarding mediation, social integration and cognition were mediated by concurrent functional outcome. However, there were no significant mediating effects of concurrent functional outcome on concurrent social integration or subsequent cognitive function. Our findings suggest there are positive relationships between social integration, functional outcome, and cognition over time. With a better understanding of these interrelationships, tailored interventions that target social integration, such as activities fostering a sense of belonging in the community and cultural diversity programs may promote improved functional and cognitive outcomes.

